# Advantages of Warm Sublimate Solutions

**Published:** 1890-07-05

**Authors:** 


### ADVANTAGES OF WARM SUBLIMATE SOLUTIONS.

Observation has shown that a sublimate solution of 1 part in 20,000 only delays the development of microbes, that 1 in 10,000 is a germicide if kept in contact with the organisms long enough; 1 in 2,000 kills bacteria in a few minutes, but to prevent the development of the spores, the solution must not be of a lower strength than 1 in 1,000, or even 1 in 500. These stronger solutions cannot, however, be injected into the uterus or pleura, or applied to extensive wounds without more or less danger from absorption, as well as of direct irritation, nor can the weaker be allowed to remain indefinitely in serous cavities with a view to obtain their full germicidal effects. But Dr. S. Ala, in a paper read before the Lancisian Society at Rome, stated that experiment in the laboratory and observation in the ward had convinced him that by using sublimate solutions at a temperature of 40 deg. C. (= 104 deg. F.) the germicidal or antiseptic action was remarkably intensified, while the local action was not, at least unfavourably. Thus a warm solution of 1 in 10,000 was as powerful a germicide as a cold one of 1 in 500, and could do no harm. A cold solution of 1 in 1,000 was less germicidal than a warm of 1 in 10,000, but the latter penetrated the tissues better, exciting to repair and accelerating the process, whereas a solution of 1 in 500 was caustic, coagulating the albumen and leaving a film which retarded the healing.

